# Examination of common culture medium for human hepatocytes and engineered heart tissue: Towards an evaluation of cardiotoxicity associated with hepatic drug metabolism *in vitro*

**DOI:** 10.1371/journal.pone.0315997

**Published:** 2024-12-23

**Authors:** Shinichiro Horiuchi, Nanae Koda, Yui Ikeda, Yuto Tanaka, Yusuke Masuo, Yukio Kato, Daiju Yamazaki

**Affiliations:** 1 Division of Pharmacology, National Institute of Health Sciences, Kawasaki, Kanagawa, Japan; 2 Faculty of Pharmacy, Kanazawa University, Kanazawa, Ishikawa, Japan; University of Iowa, UNITED STATES OF AMERICA

## Abstract

Cardiotoxicity associated with hepatic metabolism and drug–drug interactions is a serious concern. Predicting drug toxicity using animals remains challenging due to species and ethical concerns, necessitating the need to develop alternative approaches. Drug cardiotoxicity associated with hepatic metabolism cannot be detected using a cardiomyocyte-only evaluation system. Therefore, we aimed to establish a system for evaluating cardiotoxicity via hepatic metabolism by co-culturing cryopreserved human hepatocytes (cryoheps) and human iPS cell-derived engineered heart tissues (hiPSC-EHTs) using a stirrer-based microphysiological system. We investigated candidate media to identify a medium that can be used commonly for hepatocytes and cardiomyocytes. We found that the contraction length was significantly greater in the HM Dex (-) medium, the medium used for cryohep culture without dexamethasone, than that in the EHT medium used for hiPSC-EHT culture. Additionally, the beating rate, contraction length, contraction speed, and relaxation speed of hiPSC-EHT cultured in the HM Dex (-) medium were stable throughout the culture period. Among the major CYPs, the expression of *CYP3A4* alone was low in cryoheps cultured in the HM Dex (-) medium. However, improved oxygenation using the InnoCell plate increased *CYP3A4* expression to levels comparable to those found in the human liver. In addition, CYP3A4 activity was also increased by the improved oxygenation. Furthermore, expression levels of hepatic function-related gene and nuclear receptors in cryoheps cultured in HM Dex (-) medium were comparable to those in the human liver. These results suggest that the HM Dex (-) medium can be applied to co-culture and may allow the evaluation of cardiotoxicity via hepatic metabolism. Moreover, *CYP* induction by typical inducers was confirmed in cryoheps cultured in the HM Dex (-) medium, suggesting that drug–drug interactions could also be evaluated using this medium. Our findings may facilitate the evaluation of cardiotoxicity via hepatic metabolism, potentially reducing animal testing, lowering costs, and expediting drug development.

## Introduction

Animal experiments are necessary in the preclinical stage of drug development to test drug efficacy and safety. However, there is a need to reduce the number of animal experiments based on the 3Rs principle (Reduction, Refinement, Replacement) [1]. Additionally, limitations exist in predicting drug toxicity using animal models because of species differences from humans [2]. Furthermore, reduction of the costs and increasing the efficiency of drug development are necessary. Therefore, the development of an *in vitro* evaluation system using human cells is valuable.

Liver metabolism plays a major role in drug efficacy and toxicity [3, 4]. Drug-induced cardiotoxicity is a major cause of drug withdrawal from the market or discontinuation of development [5, 6], and several drugs whose toxicity is related to drug–drug interactions have been withdrawn due to cardiotoxicity [7–9]. For example, terfenadine, a human ether-a-go-go related gene (hERG) channel blocker, induces torsades de pointes and ventricular arrhythmias by prolonging the QT interval, whereas fexofenadine, which is a metabolite of terfenadine mediated by CYP3A4, has hERG channel blocking activity 1/20^th^ that of terfenadine [10]. Therefore, the cardiotoxicity risk of terfenadine is extremely low when administered alone but increases when administered in combination with CYP3A4 inhibitors such as cefaclor, ketoconazole, and medroxyprogesterone [11]. Similarly, while cyclophosphamide itself has limited cardiotoxic effects, its metabolite acrolein is primarily responsible for inducing cardiotoxicity [12, 13]. These findings highlight the critical role of drug metabolism in modulating cardiotoxicity. Therefore, liver drug metabolism should be considered when evaluating drug cardiotoxicity. Drug cardiotoxicity and hepatic drug metabolism are currently evaluated separately *in vitro* [14, 15]. Limitations exist in predicting cardiotoxicity risk from individual evaluations owing to the complexity of hepatic drug metabolism [16]. Therefore, evaluation of cardiotoxicity associated with hepatic drug metabolism in a co-culture system of cardiomyocytes and hepatocytes may improve risk prediction.

Cell culture devices such as microphysiological systems (MPS) are being actively developed as evaluation systems that mimic *in vivo* conditions [17–20]. Some methods allow inter-organ evaluation by connecting culture compartments for cells derived from different organs and perfusing them with culture medium [21–23]. We aimed to construct an *in vitro* system to evaluate cardiotoxicity associated with hepatic drug metabolism by co-culturing cryopreserved human hepatocytes (cryoheps) and human iPS cell-derived engineered heart tissue (hiPSC-EHTs) using an MPS [24, 25]. A key challenge in co-culturing cells from different organs is the need for distinct culture media tailored to each cell type’s requirements. For in vitro evaluation of cardiotoxicity associated with hepatic drug metabolism, a culture medium compatible with both hepatocytes and cardiomyocytes is essential. Therefore, in this study, we systematically investigated the common medium for hepatocytes and cardiomyocytes (HCMM: Hepatocyte-Cardiomyocyte Maintenance Medium). An effective HCMM should support optimal functionality in both cardiomyocytes and hepatocytes. To ensure this, we evaluated the contractile properties of hiPSC-EHTs and the expression of major *CYPs* in cryoheps across the candidate media, to identify a suitable HCMM. Hepatocytes adopt a hypoxic state when cultured on common plastic plates [26]. Therefore, we examined the effect of oxygen concentration on CYP expression using a culture vessel with an oxygen-permeable membrane [27]. We also report the effects of oxygenation on CYP expression using cryoheps.

## Materials and methods

### Culture medium

Hepatocyte Maintenance medium (HM medium) used to culture cryoheps, MH medium without dexamethasone (HM Dex (-) medium), and EHT medium used to hiPSC-EHT were examined as candidate HCMM. Dexamethasone was excluded from the HM medium because it is known to influence action potential and heart contraction [28, 29].

### Cryopreserved human hepatocyte culture

Cryopreserved human hepatocytes (Lot #3–51) were obtained from XenoTech (Lenexa, KS, USA). The cells were thawed using OptiThaw (XenoTech) and suspended at 7.2 × 10^5^ cells/mL using OptiPlate (XenoTech). Subsequently, 500 μL of the cell suspension was seeded on a collagen-coated 24-well polystyrene (PS) plate or InnoCell plate. After 4–5 h, the medium was replaced with 500 μL of HM medium. The medium was replaced with HM medium, HM Dex (-) medium, or EHT medium on the following day and cultured for 72 h. The compositions of these media are shown in S1 Table. The InnoCell plate used in this study is a 24-well plate with an oxygen-permeable membrane on the cell attachment surface (https://jp.mitsuichemicals.com/en/special/innocell/index.htm).

### Stromal cell subculture

The bone marrow stromal cell line HS27a was obtained from the American Type Culture Collection (Rockville, MD, USA) [30]. HS27a cells were cultured in gelatin-coated 10-cm dishes in Dulbecco’s modified Eagle’s medium (DMEM; Gibco BRL, Paisley, Scotland) supplemented with 10% FBS (Cytiva, Tokyo, Japan), GlutaMAX (Gibco), MEM non-essential amino acids (Gibco), and penicillin–streptomycin solution (Fujifilm Wako), and passaged once a week.

### Creating human iPS cell-derived engineered heart tissue

Molds were prepared by adding 2 mL 2% agarose in PBS (Invitrogen, Carlsbad, Calif., USA) to each well of a 24-well plate and placing spacers (C0002, EHT Technologies, Hamburg, Germany). After the agarose solidified, the spacer was removed, and PDMS racks (C0001, EHT Technologies) were placed into each agarose mold [24, 25]. HS27a cells were collected from a 10-cm dish by trypsinization. iCell cardiomyocyte 2.0 (FUJIFILM Cellular Dynamics, Inc., Madison, WI, USA) was thawed according to the manufacturer’s instructions. HS27a cells and iCell cardiomyocytes^2^ were mixed at a ratio of 1:10 and centrifuged (200 ×*g*, 5 min, 23°C). The supernatant was discarded and the pellet was suspended in an iCell maintenance medium (FUJIFILM Cellular Dynamics, Inc.) at a final concentration of 5 mg/mL fibrinogen (Sigma-Aldrich, St Louis, MO, USA) and 3 U/mL thrombin (Sigma-Aldrich). The suspension contained 5.0 × 10^4^ cells of HS27a and 5.0 × 10^5^ cells of iCell cardiomyocyte ^2^ per 100 μL. The suspension was then poured into an agarose mold. The constructs were incubated for 90 min at 37°C and 5% CO_2_ for fibrinogen polymerization. The polymerized hiPSC-EHTs were transferred to the wells of a 24-well plate containing a 2 mL iCell maintenance medium.

### Human iPS cell-derived engineered heart tissue culture

hiPSC-EHTs were cultured in iCell cardiomyocyte maintenance medium for 3 weeks and in EHT medium for over 1 week [31]. Thereafter, the medium was replaced with HM medium or HM Dex (-) medium, and contractile properties were observed.

### Evaluation of contractile properties in human iPS cell-derived engineered heart tissue

Contraction of hiPSC-EHTs was recorded for 10 s using a microscope CKX41 (OLYMPUS, Tokyo, Japan) equipped with a high-speed camera HAS-U2 (DETECT, Tokyo, Japan) (resolution: 1920 × 1080, number of frames: 60 fps, shutter speed: 1/700). The recorded moving images were analyzed using SI8000 software (SONY, Tokyo, Japan) and a versatile open software tool MUSCLEMOTION [32].

### RNA isolation

The cultured cells were washed twice with PBS. hiPSC-EHTs were treated with proteinase K (Thermo Fisher Scientific, Waltham, MA, USA) at a final concentration of 0.22 mg/mL for 10 min at 55°C before RNA isolation. Total RNA was then isolated from the cells using the RNeasy® Total RNA Extraction Kit (Qiagen, Hilden, Germany) according to the manufacturer’s instructions.

### Real-time polymerase chain reaction

Total RNA was reverse transcribed to cDNA using total RNA and high-capacity RNA-to-cDNA (Thermo Fisher Scientific) according to the manufacturer’s instructions. Gene expression in hepatocytes was measured using a QuantStudio 7 Flex Real Time PCR System (Applied Biosystems, Foster City, CA, USA) with TaqMan Fast Advanced Master Mix (Applied Biosystems). The primer and probe sets used are shown in S2 Table. Pooled RNA derived from human liver (BioChain Institute, Inc.) was used to generate the standard curve, and the expression level was set to one. The relative expression levels were calculated using the line equation for the standard curve. Gene expression in hiPSC-EHTs was measured using the QuantStudio 7 flex Real Time RCR System and THUNDERBIRD® Next SYBR® qPCR Mix (TOYOBO, Osaka, Japan). The primers used are shown in S3 Table. RNA derived from the left ventricle (BioChain Institute, Inc.) was used to generate the corresponding standard curve, and the expression level was set to one. The relative expression levels were calculated using the line equation for the standard curve.

### CYP induction in cryoheps

Cryoheps were cultured for 48 h from the second day of seeding and exposed to 20 μM rifampicin (Fujifilm Wako Pure Chemicals Co., Osaka, Japan), 50 μM omeprazole (Fujifilm Wako), and 500 μM phenobarbital (Fujifilm Wako). 0.1% DMSO (Sigma-Aldrich) was used as a vehicle.

### CYP enzyme metabolic activity quantification in cryoheps

Cryoheps were incubated in HM Dex (-) medium containing a cocktail of CYP probe substrates (phenacetin 20 μM (for CYP1A2), diclofenac 1 μM (for CYP2C9), mephenytoin 40 μM (for CYP2C19), bufuralol 5 μM (for CYP2D6), midazolam 2 μM (for CYP3A)) at 37°C. After incubation for 60 min, the incubation media was collected and kept at -80°C until LC–MS/MS analyses. The metabolites formed by the CYP probe cocktail were quantified by LC-MS/MS using a Nexera X2 LC system coupled with an LCMS-8050 (Shimadzu, Kyoto, Japan). The detected mass numbers and collision energy (CE) were as follows; acetaminophen for CYP1A2 (152.0 > 110.0, CE: − 9 V), CE: − 24 V), 4′-hydroxydiclofenac for CYP2C9 (312.0 > 230.0, CE: − 32 V), 4′-hydroxymephenytoin for CYP2C19 (276.2 > 235.1, CE: − 17 V), 1′-hydroxybufuralol for CYP2D6 (278.0 > 186.0 CE: − 19 V), 1′-hydroxymidazolam for CYP3A4 (342.0 > 203.0, CE − 27 V) [33].

### Statistical analysis

Comparisons between two groups were performed using Student’s t-test, assuming equal or unequal variances based on the f-test with two-tailed, in Excel.

## Results

### Contractile properties in human iPS cell-derived engineered heart tissue

**Fig 1:**
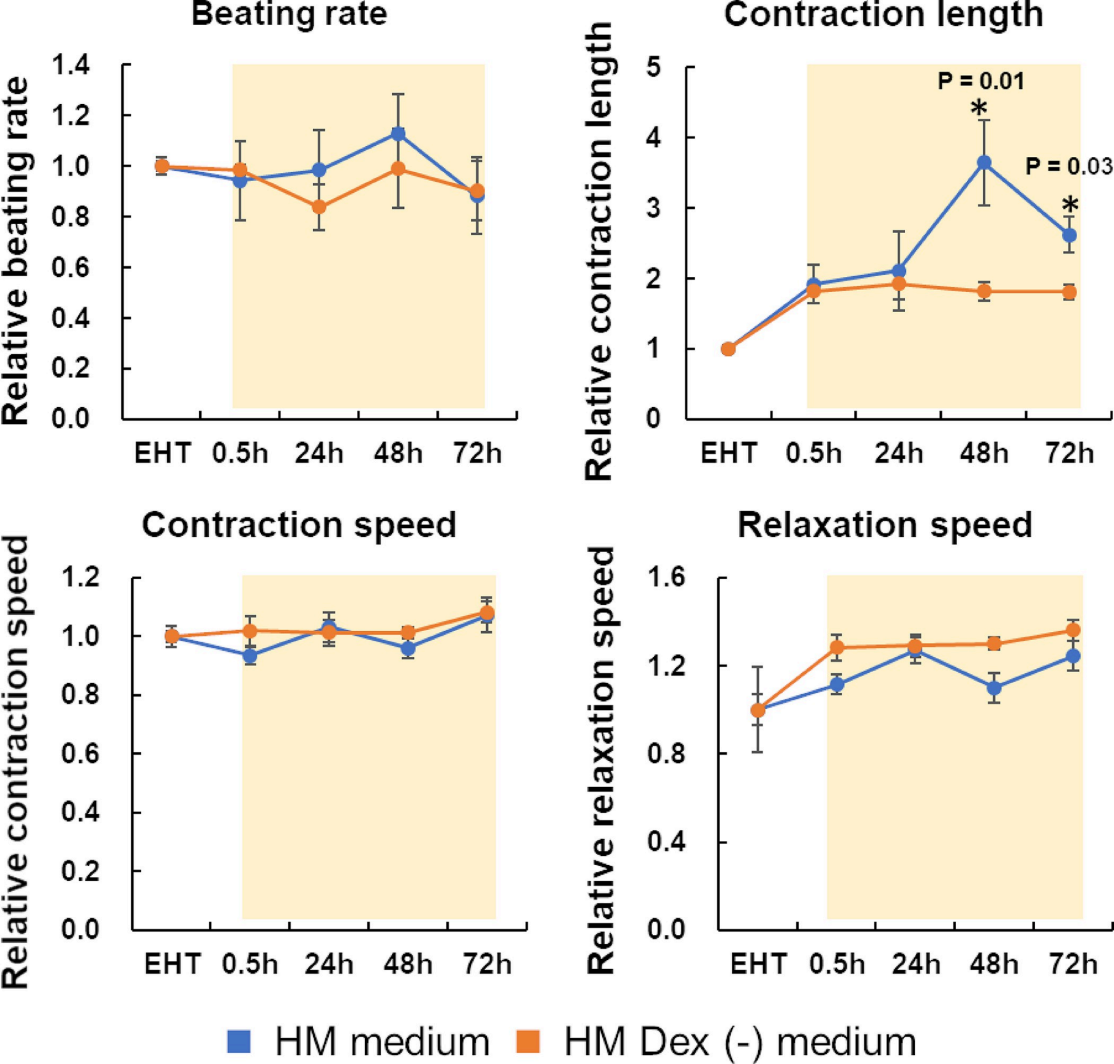
Contractile properties of human iPS cell-derived engineered heart tissues (hiPSC-EHTs). hiPSC-EHTs were cultured in EHT medium for contractile property analysis and then cultured in the HM or HM Dex (-) media. Thereafter, the contractile properties of hiPSC-EHTs were analyzed after 0.5, 24, 48, and 72 h of culture. Movie images were analyzed using SI8000 software. The graph bar shows the average mean, and the error bar shows the standard error (n = 4). * Statistically significant change compared to the 0.5 h values (p < 0.05).

Contractile properties (beating rate, contraction length, contraction speed, and relaxation speed) were observed in three candidate media to evaluate hiPSC-EHT functions. The contractile properties of the hiPSC-EHTs were evaluated based on those in the EHT medium, the choice medium for culturing hiPSC-EHTs. The contraction length exhibited a notable increase following the transition from the EHT medium (dedicated to hiPSC-EHTs) to both the HM and HM Dex (-) media (Fig 1). The other parameters did not decrease upon transition to the HM and HM Dex (-) media. After the transition to the HM medium, the contraction length increased until 48 h and then decreased. However, all parameters were stable throughout the culture period in the HM Dex (-) medium. These results suggest that the HM Dex (-) medium increases the contraction length and stabilizes all parameters, and is the most suitable for hiPSC-EHT culture, of the three candidate media.

### Expression of myocardial-specific genes in human iPS cell-derived engineered heart tissue

**Fig 2:**
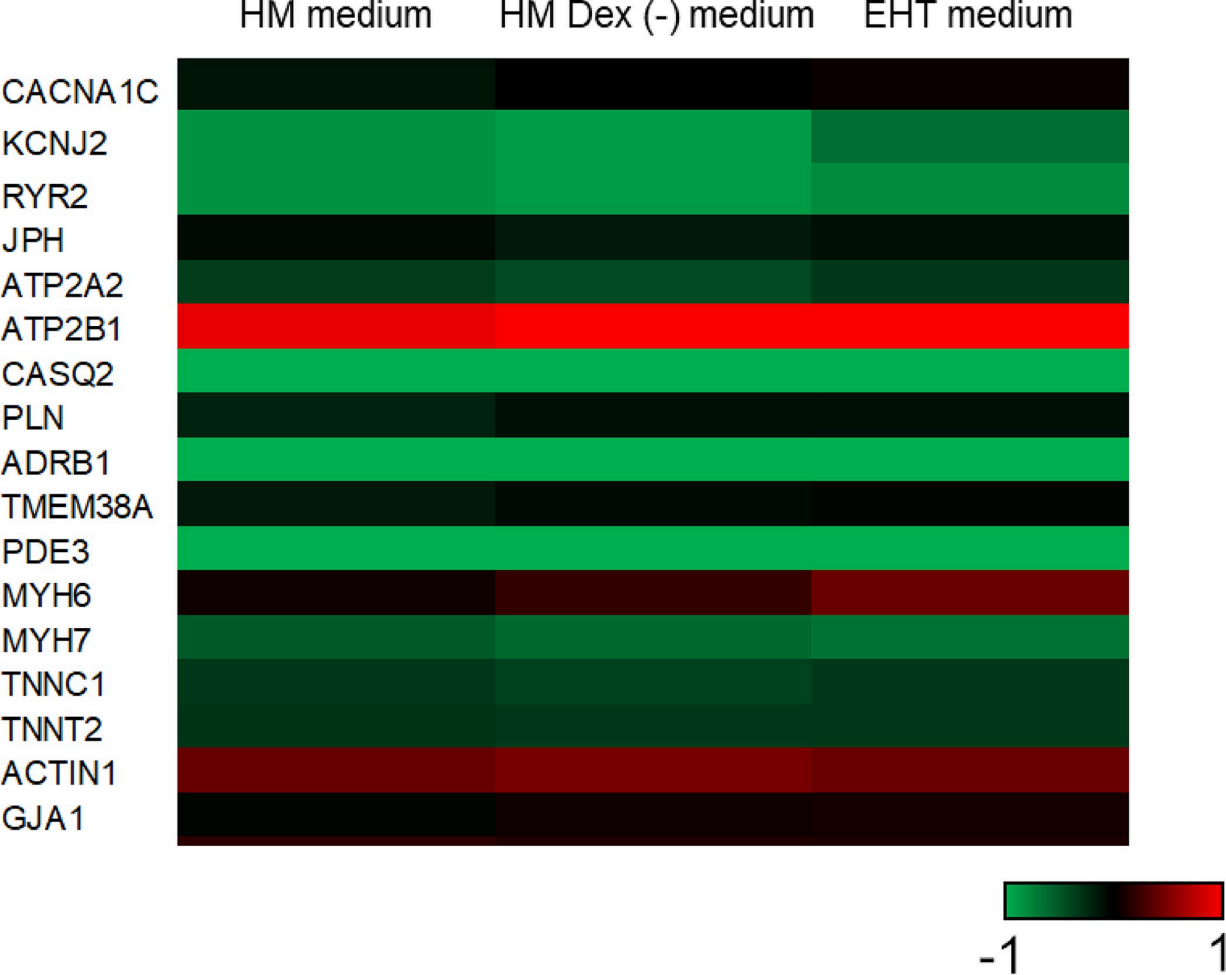
Heatmap showing cardiomyocyte-specific gene expression in human iPS cells-derived engineered heart tissues (hiPSC-EHTs). hiPSC-EHTs were cultured in EHT medium for over 1 week and then in EHT medium, HM medium, or HM Dex (-) medium for 72 h. RNA was collected from the tissues at the endpoint and analyzed using qPCR (n = 4). The color of the Heatmap shows the value of log2 (relative expression level compared to the human left ventricle).

We measured the expression levels of 17 myocardial-specific genes to compare the characteristics of hiPSC-EHTs cultured in the 3 media. The difference in the expression levels of 16 genes, except *MYH6*, was within 2-fold across the 3 media (Fig 2 and S1 Fig). The expression level of *MYH6* was 2.2 times lower in the HM than in the EHT medium. Additionally, principal component analysis based on the expression levels of 17 genes showed that the contribution rate of the first component was approximately 99%, with similar eigenvalues across the three candidate media (S2 Fig). These results suggest no significant differences in the myocardial-specific properties of hiPSC-EHTs among the three candidate media.

### CYP expression in cryopreserved hepatocytes

**Fig 3:**
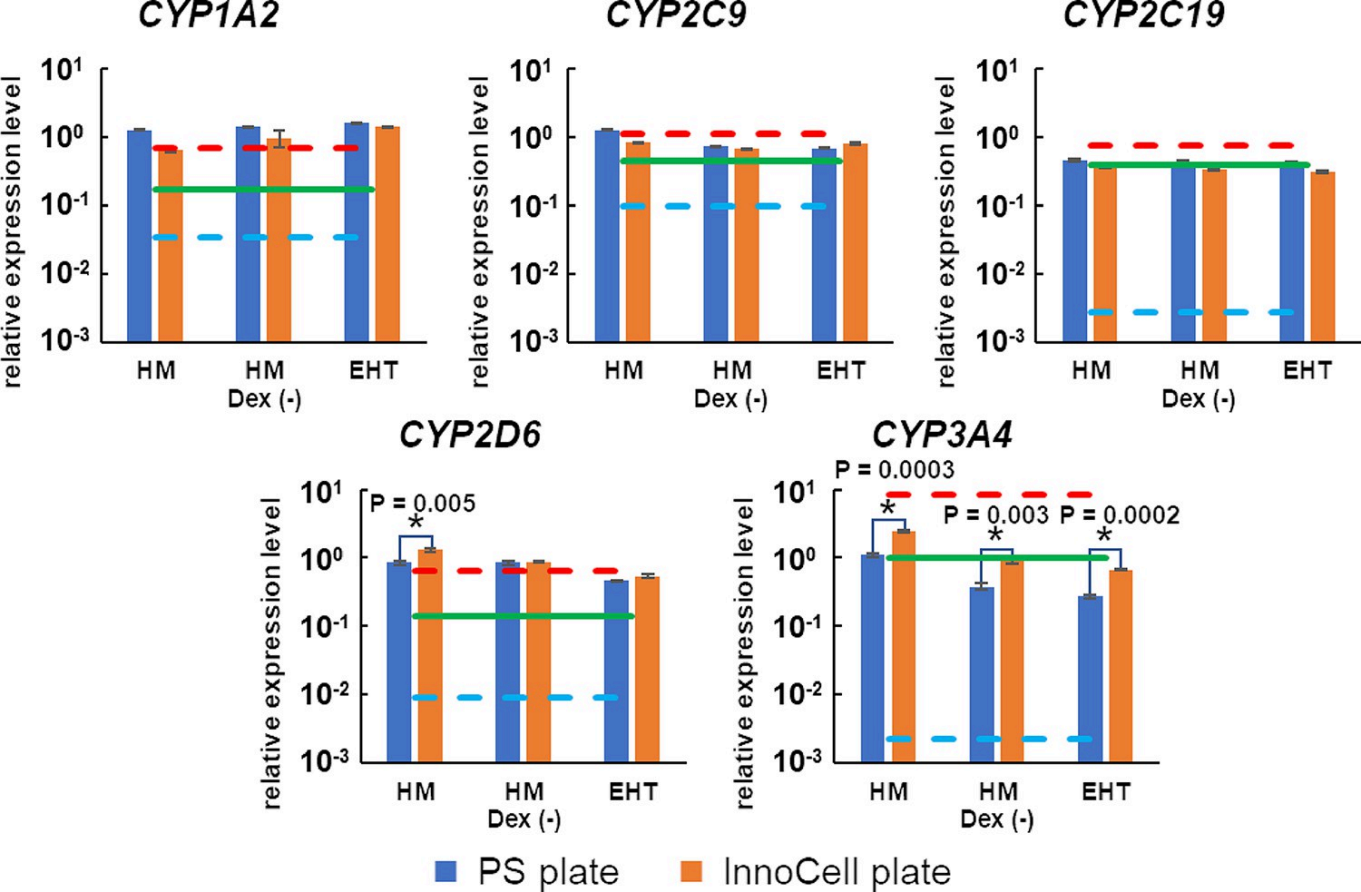
Cytochrome P450 expression in cryopreserved human hepatocytes (cryoheps). Cryoheps were cultured in EHT medium, HM medium, or HM Dex (-) medium for 72 h from the day of seeding. Polystyrene (PS) plates and InnoCell plates were used for the culture. RNA was collected from the cells at the endpoint and analyzed by qPCR. The graph bar shows the mean of the relative expression levels compared to the human liver, and the error bar shows the standard error (n = 3). The green, red, and blue lines show the mean, maximum, and minimum values of expression in the 22 lots of cryoheps under vendor-recommended conditions [34]. * Statistically significant increase in the InnoCell plate compared with the values in the PS plates (p < 0.01).

The expression of major *CYPs*, phase I drug metabolizing enzymes, was observed in the three candidate media to evaluate cryoheps function. *CYPs* expression in cryoheps was evaluated based on that in the HM medium. The expression of *CYPs* other than *CYP3A4* in the cryoheps did not differ by more than 2-fold among the three candidate media (Fig 3). However, *CYP3A4* expression was more than 2-fold lower in the EHT and HM Dex (-) media than that in the HM medium. This result suggests that dexamethasone significantly contributes to the expression of *CYP3A4* in the HM medium. The *CYP* expression levels were compared to the previously reported average values of 22 lots of cryoheps under vendor-recommended conditions to evaluate the adequacy of drug metabolism ability [34]. The expression level of *CYP3A4* alone was lower in the HM Dex (-) and EHT media than the average value of 22 lots of cryoheps under vendor-recommended conditions. In addition, we attempted to improve *CYP* expression using an InnoCell plate, in which the culture bottom is an oxygen-permeable membrane. When cultured on InnoCell plates, the expression level of *CYP3A4* in the EHT and HM Dex (-) media increased to approximately 80% of the expression observed in cells cultured in HM medium on PS plates and was comparable to the average value of 22 lots of cryoheps under the vendor-recommended conditions. These results suggest that cryoheps may show sufficient drug metabolic activity even in the EHT and HM media using InnoCell plates.

### Expression of hepatic function-related genes in cryopreserved hepatocytes

**Fig 4:**
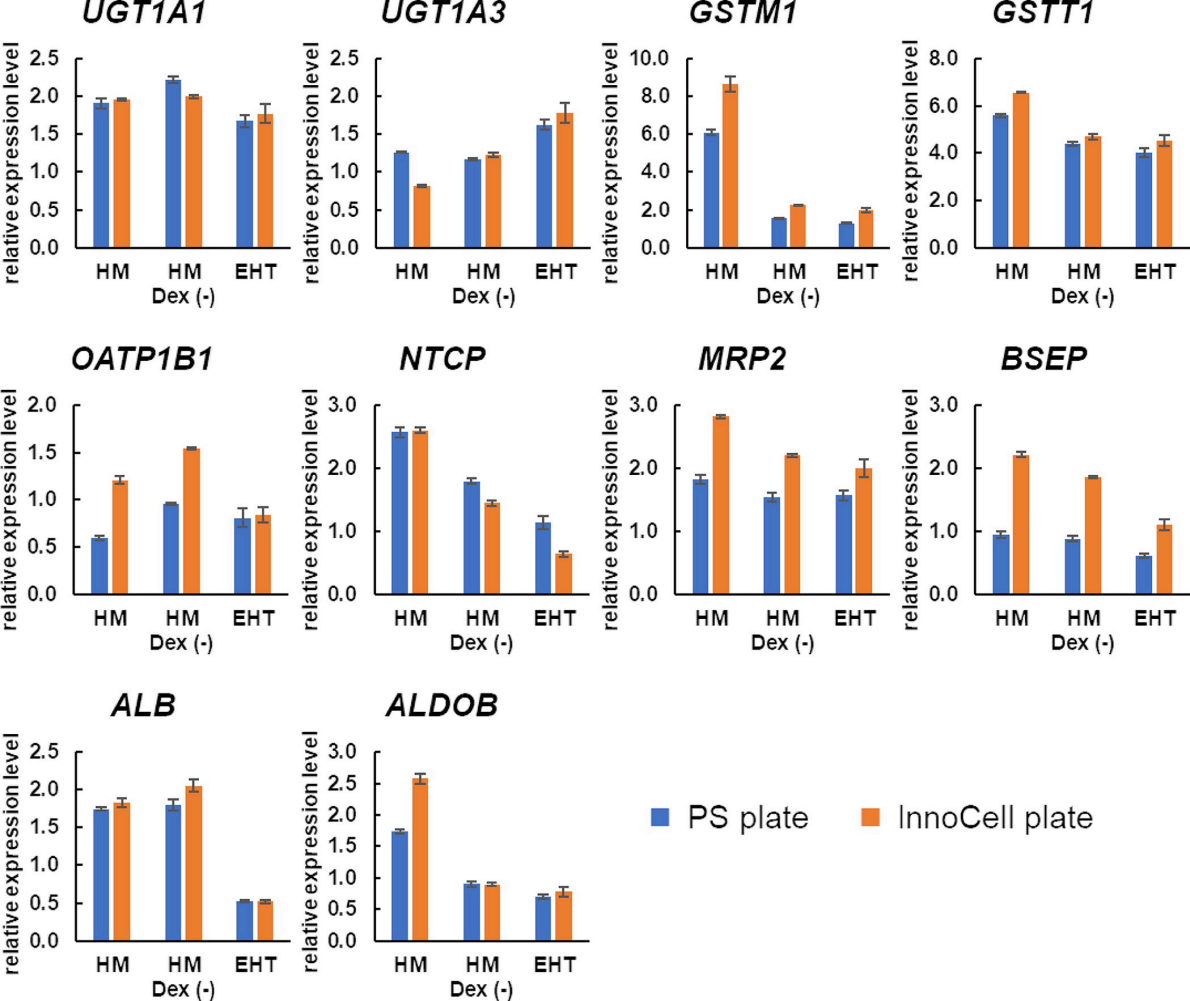
Expression of genes related to liver function in cryopreserved human hepatocytes (cryoheps). Cryoheps were cultured in the EHT medium, HM medium, or HM Dex (-) medium for 72 h from the day of seeding. PS plates and InnoCell plates were used for the culture. RNA was collected from the cells at the endpoint and analyzed by qPCR. The graph bar shows the average mean of the relative expression levels compared with the human liver, and the error bar shows the standard error (n = 3).

We also determined the expression levels of hepatic function-related genes other than *CYPs*. When dexamethasone was removed from the HM medium, which is the choice medium for culturing hepatocytes, the expression of *GSTM1* alone was reduced by half or less (Fig 4). However, the expression level of *GSTM1* in the HM Dex (-) medium was higher than 1, relative to that in the human liver. Furthermore, the expression levels of other hepatic function-related genes in the HM Dex (-) medium were comparable to those in the human liver (relative expression = 1). These results suggest that hepatic function-related genes other than *CYPs* are sufficiently expressed in the HM Dex (-) medium. Additionally, the expression of biliary excretion transporters, such as *MRP2* and *BSEP*, trended to increase in InnoCell cultures.

### Expression of nuclear receptors in cryopreserved hepatocytes

**Fig 5:**
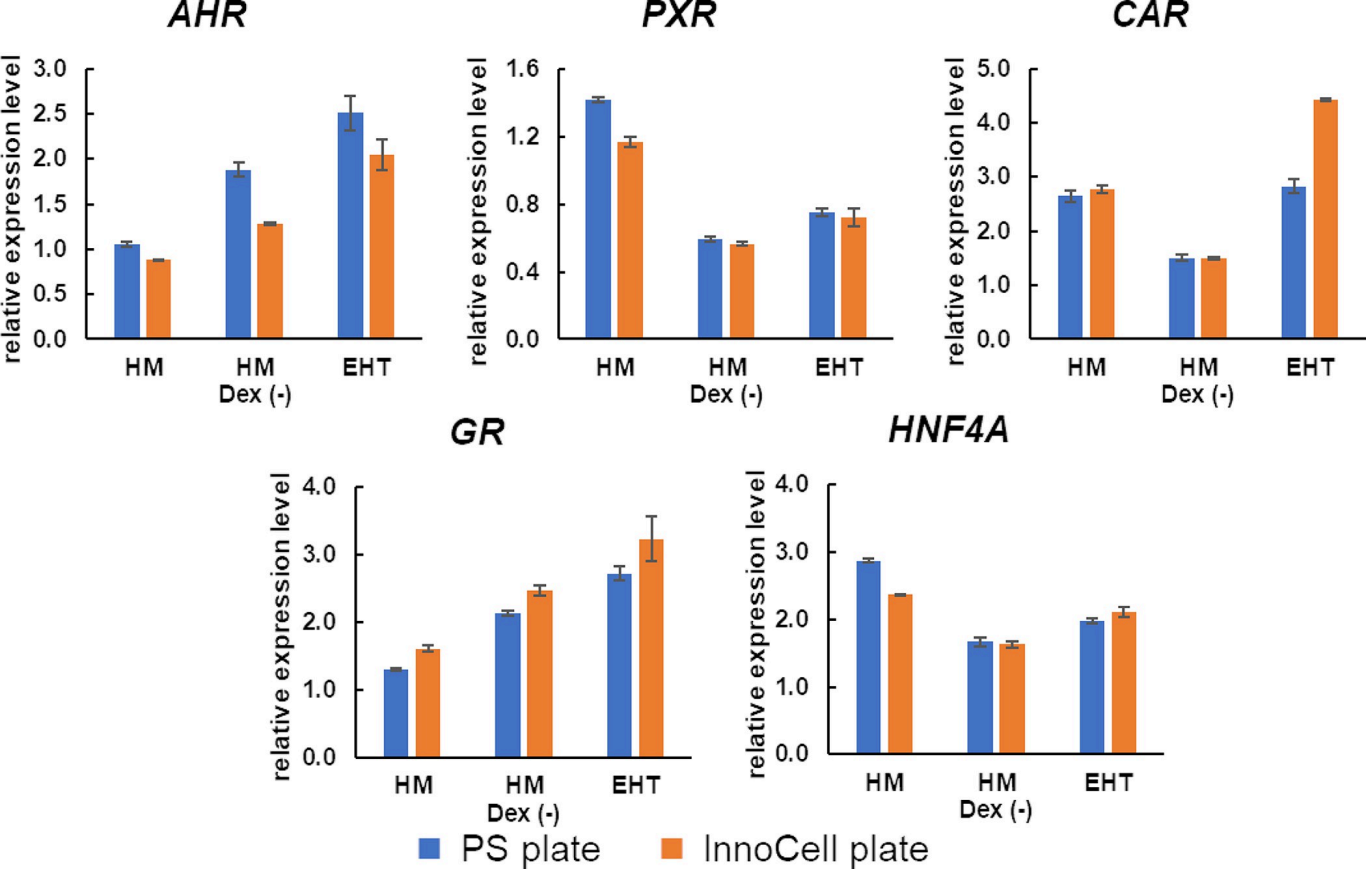
Expression of nuclear receptors in human cryopreserved hepatocytes (cryoheps). Cryoheps were cultured in the EHT medium, HM medium, or HM Dex (-) medium for 72 h from the day of seeding. Polystyrene plates and InnoCell plates were used for the culture. RNA was collected from the cells at the endpoint and analyzed using qPCR. The graph bar shows the average mean of the relative expression levels compared with the human liver, and the error bar shows the standard error (n = 3).

The evaluation of contraction in hiPSC-EHTs and the expression of hepatic function-related genes, including *CYPs*, in cryoheps, suggests the HM Dex (-) medium as the most fitting HCMM. Nuclear receptors are activated by exposure to compounds, such as drugs, which regulate the gene expression of drug-metabolizing enzymes and drug transporters. Therefore, nuclear receptors are crucial factors in the evaluation of drug–drug interactions. The expression levels of the nuclear receptors except *PXR* were higher in the HM Dex (-) medium, our primary candidate HCMM under consideration, in the human liver (Fig 5). Additionally, the expression level of *PXR* was 0.6 relative to that in the human liver, showing no difference in order of magnitude.

### CYP induction by typical inducers in cryopreserved hepatocytes

**Fig 6:**
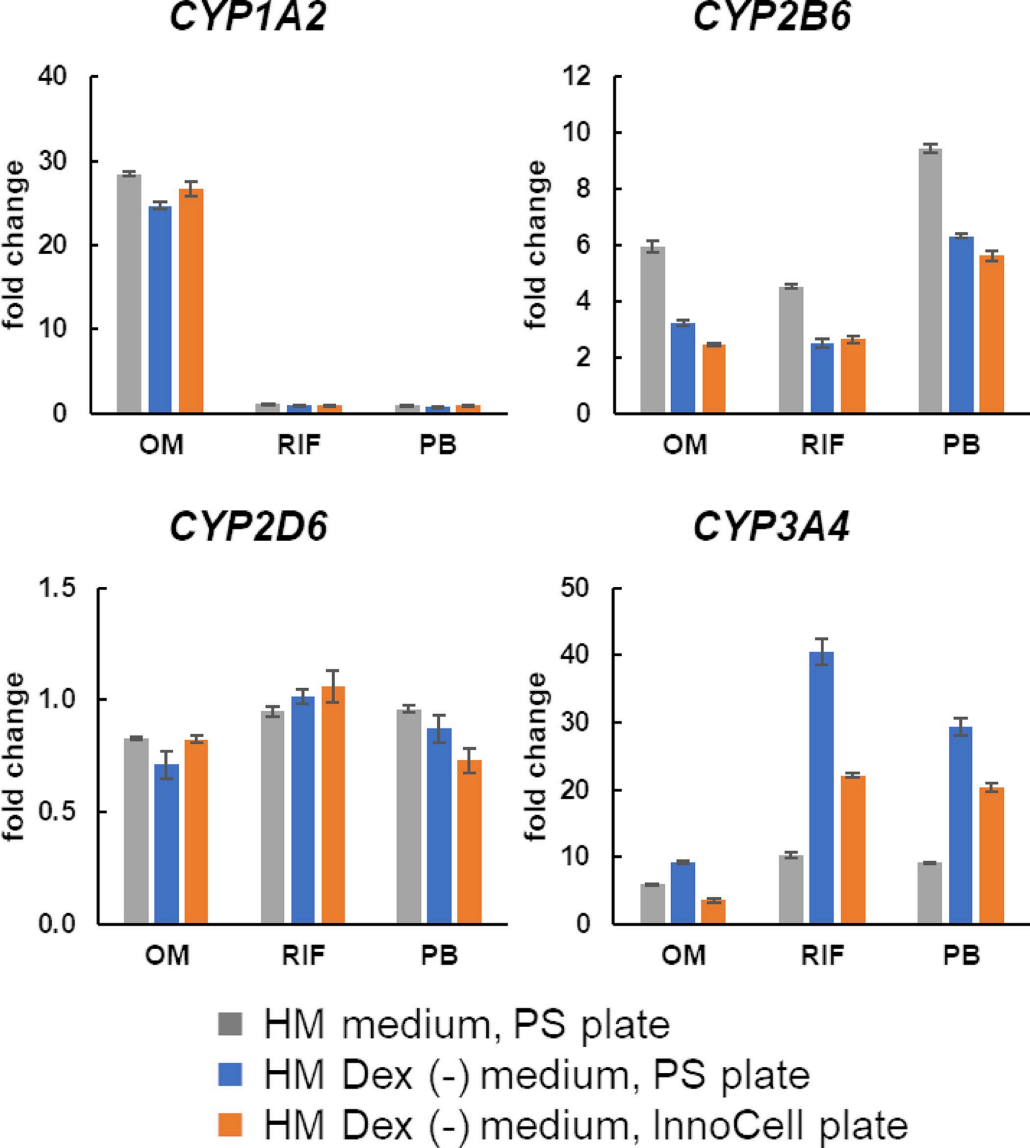
Induction of cytochrome P450 expression in human cryopreserved hepatocytes (cryoheps). Cryoheps were cultured in the HM or HM Dex (-) media with inducer for 48 h from day 2 after seeding. Polystyrene and InnoCell plates were used for the culture. RNA was collected from the cells at the endpoint and analyzed by qPCR. The graph bar shows the average mean gene expression fold change upon exposure to the inducer (OM: Omeprazole, RIF: Rifampicin, PB: Phenobarbital), and the error bar shows standard error (n = 3).

CYP induction in cryoheps is required to evaluate drug–drug interactions. Therefore, we examined CYP induction by a typical inducer in cryoheps cultured in the HM Dex (-) medium, our primary candidate HCMM. We observed *CYP1A2* induction by omeprazole, *CYP2B6* and *CYP3A4* induction by phenobarbital, and *CYP3A4* induction by rifampicin in cultures using both the PS and InnoCell plates (Fig 6). Furthermore, the fold-changes after induction were comparable to those achieved in cryoheps cultured in the HM medium on PS plates.

### CYP activity in cryopreserved hepatocytes

**Fig 7:**
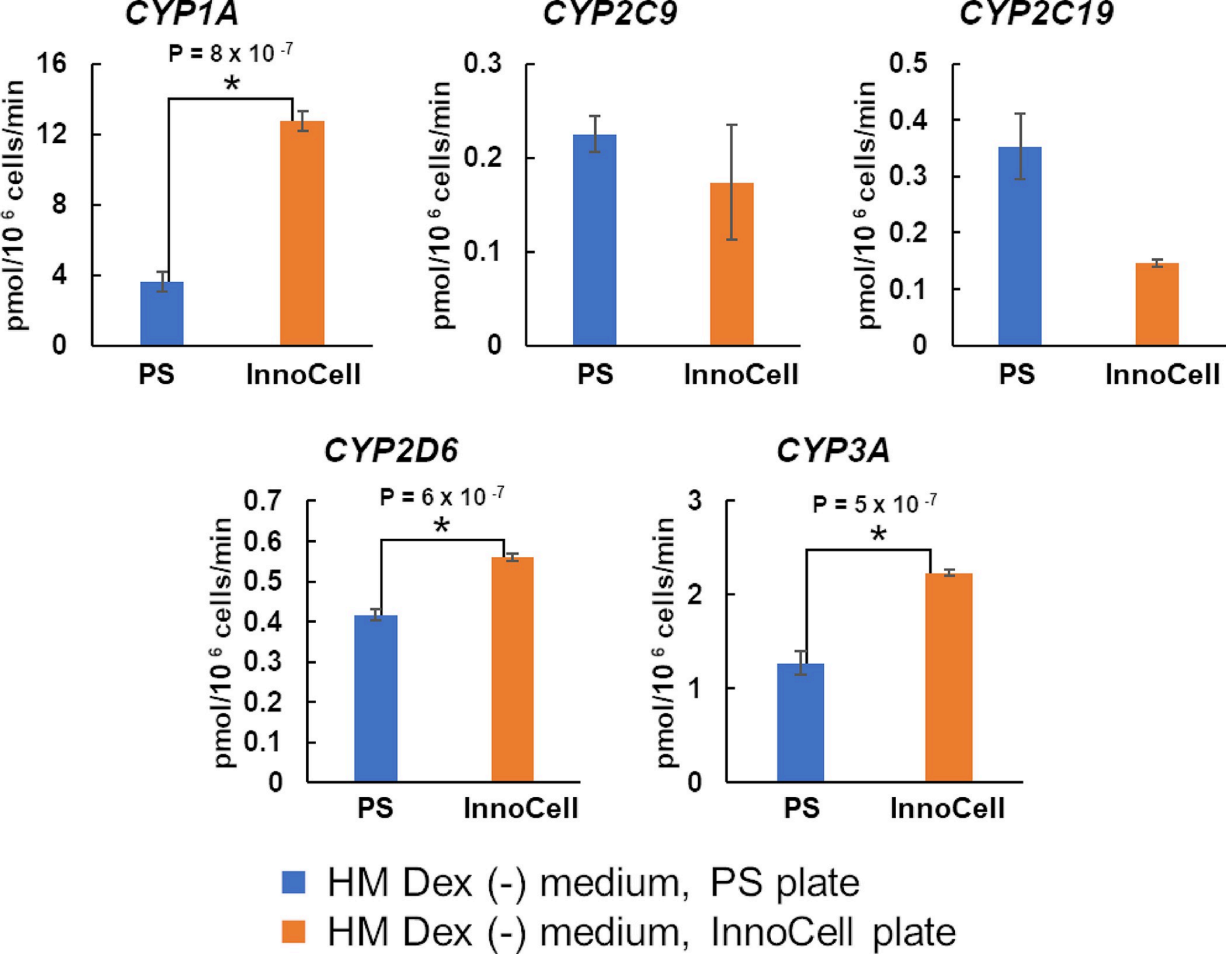
Cytochrome P450 (CYP) activity in human cryopreserved hepatocytes (cryoheps). Cryoheps were cultured in the HM Dex (-) medium on polystyrene (PS) or InnoCell plates for 72 h from the day of seeding. PS and InnoCell plates were used for the culture. Cryoheps were incubated in the HM medium Dex (-) containing a cocktail of CYP probe substrates. After 60 min of incubation, the incubation media were collected and the metabolites were measured using LC-MS/MS. The graph bar shows the average mean CYP activity and the error bar shows the standard error (n = 4). * Statistically significant increase in the InnoCell plate compared to the values in the PS plates (p < 0.01).

Based on the contractile properties of hiPSC-EHTs and CYPs expression in cryoheps, we selected the HM Dex (-) medium as the HCMM. Subsequently, we compared the activity of CYPs in cryoheps cultured in HM Dex (-) medium between PS and InnoCell plates. The activities of CYP1A, CYP2D6, and CYP3A were significantly enhanced when cryoheps were cultured on InnoCell plates (Fig 7).

## Discussion

Drug metabolism in the liver affects the efficacy and toxicity of drugs, and some drugs have been withdrawn from the market because of drug-induced cardiotoxicity associated with hepatic drug metabolism [8]. Therefore, consideration of the effects of hepatic drug metabolism is crucial for evaluating the cardiotoxicity risk of drugs. Individual evaluations of hepatic drug metabolism and cardiotoxicity have limitations in risk prediction owing to the complexity of hepatic drug metabolism [35]. Therefore, the construction of a risk evaluation system based on the coculture of hepatocytes and cardiomyocytes is required. Hence, we aimed to construct a co-culture evaluation system using cryoheps and hiPSC-EHTs based on our established contractile property-based cardiotoxicity evaluation system using hiPSC-EHTs. An ideal co-culture medium, in which both cryoheps and hiPSC-EHTs function effectively, is crucial for successfully establishing such an evaluation system. Therefore, in this study, we evaluated different media to identify a common medium suitable for both cryoheps and hiPSC-EHTs. EHT medium, a medium of choice for culturing hiPSC-EHTs; HM medium, a medium for culturing cryoheps; and HM Dex (-) medium, an HM medium without dexamethasone, were assessed as candidate HCMM. Large changes in contraction are advantageous for evaluating the contraction of hiPSC-EHTs. The contraction length was significantly larger in the HM and HM Dex (-) media than in the EHT medium, which is a dedicated culture medium for hiPSC-EHTs. This difference may be due to varying Ca^2^⁺ concentrations in the media. The HM and HM Dex (-) media are based on William’s E medium with 1.8 mM Ca^2^⁺, whereas EHT medium is based on RPMI medium with 0.4 mM Ca^2^⁺. Hansen et al. reported that the contraction force in the EHT medium is Ca^2+^-concentration dependent [25]. This suggests that the contraction length for 3D cardiac tissue was larger in the HM or HM Dex (-) media because of the higher Ca^2+^ concentration, compared with that in the EHT medium. Moreover, we confirmed that the EHT medium with a Ca^2+^ concentration adjusted to 1.8 mM was not suitable as an HCMM because of cryohep detachment. Additionally, stable contraction properties are important for a robust evaluation. While changes in contraction length were observed over time in the HM medium, all parameters remained stable until 72 h in the HM Dex (-) medium. Furthermore, the expression of 17 cardiac-specific genes indicated no significant differences in the cardiac characteristics of hiPSC-EHTs cultured in the three candidate media. Moreover, arrhythmia caused by paliperidone and contraction suppression and arrhythmia caused by terfenadine were detected in hiPSC-EHTs cultured in HM Dex (-) medium (S3 Fig), as previously reported [36, 37]. Therefore, we conclude that the HM Dex (-) medium is the most suitable medium for evaluating contraction properties in hiPSC-EHTs.

Although many enzymes are involved in drug metabolism, CYPs are primarily involved in the metabolism of approximately 75% of the drugs [38]. CYP3A4, CYP2D6, CYP2C9, CYP2C19, and CYP1A2 are the most important CYPs enzymes, accounting for approximately 60% of the total amount of CYPs in the liver [39, 40]. In this study, we evaluated the drug-metabolizing abilities of cryoheps cultured in three candidate media by comparing the expression levels of *CYP3A4*, *CYP2D6*, *CYP2C9*, *CYP2C19*, and *CYP1A2*. The difference in the expression of *CYPs* other than *CYP3A4* was less than 2-fold among the three candidate media. Furthermore, the expression levels of these *CYPs* were the same as the average value of 22 lots of cells under the conditions recommended by the vendor. The expression level of *CYP3A4* was more than 2-fold lower when dexamethasone was removed from the HM medium. Dexamethasone has been reported to suppress the expression of *IL6* [41], which negatively regulates *CYP3A4* expression [42]. In this study, *IL6* expression was increased when dexamethasone was removed (S4 Fig), suggesting that the decreased expression of *CYP3A4* in HM Dex (-) medium is mediated by the elevated IL6 levels. However, the expression level of *CYP3A4* in the HM Dex (-) medium showed a notable increase, reaching approximately 80% of the expression observed in cells cultured in the HM medium on PS plates by optimizing the oxygen supply to the cells using InnoCell plates. The expression level was similar to the average value of 22 lots of cells cultured under the conditions recommended by the vendor. These results suggest that sufficient metabolic activity can be obtained using the HM Dex (-) medium by improving oxygen supply. The next step is to demonstrate that the test drugs are metabolised in the HM Dex(-) medium as much as in the HM medium, in order to evaluate cardiotoxicity via hepatic metabolism. We are considering the use of the kinetic pump-integrated microfluidic plate (KIM-Plate) developed by Kimura et al. [43] as a culture vessel for co-culturing cryoheps and hiPSC-EHTs. This device has a microstirrer installed in the microchannel between two wells to allow medium perfusion. The device is being studied to replace the plastic bottom of the normal case with an oxygen-permeable membrane, such as that used in InnoCell plates, to improve the oxygen supply. An improved oxygen supply will allow us to obtain results that more closely reflect drug metabolism.

An optimized HCMM is essential to ensure optimal functionality for cryoheps and hiPSC-EHT. The HM Dex (-) medium is the most suitable for hiPSC-EHTs because the contraction length is larger than that achieved with the EHT medium, which is a medium for cardiomyocytes. A longer contraction length is advantageous in detecting more definite negative inotropic action. Moreover, the contraction length, contraction speed, relaxation speed, and number of beats are stable during 72 h of culture in the HM Dex (-) medium. In contrast, contraction parameters of hiPSC-EHTs cultured in HM medium with dexamethasone were more unstable than those in HM Dex(-) medium, especially the contraction length, which drastically increased until 48 h and then decreased. Therefore, dexamethasone should be removed from HM medium for stable cardiotoxicity evaluation. The expression of *CYP3A4* was significantly lower in cryoheps cultured in the HM Dex (-) medium than in those cultured in the HM medium, a medium for hepatocytes; however, the expression was restored with the use of an InnoCell plate. In addition, when cryoheps were cultured in HM Dex (-) medium on InnoCell plates, the expression levels of all *CYP* genes were comparable to or higher than the average value of 22 lots of cryoheps under optimal conditions. Moreover, the expression levels of phase II drug metabolizing enzymes and bile acid uptake/excretion transporters in the HM Dex (-) medium were one or more relative to the liver. These results reinforce that the HM Dex (-) medium is the most optimal HCMM.

Drug–drug interactions in the liver have been reported to affect drug toxicity. Terfenadine can cause cardiac arrhythmias when used in combination with drugs that inhibit CYP3A4 activity [44]. Acetaminophen can cause hepatotoxicity when used in combination with rifampicin by increasing the metabolite NAPQI [45]. This reaction involves the induction of CYP by rifampicin. Therefore, the ability to induce CYPs is important when evaluating drug–drug interactions. In addition, drugs induce CYPs by activating the nuclear receptor [46, 47]. The expression of nuclear receptors comparable to liver levels and the induction of CYP expression by typical inducers were confirmed in the HM Dex (-) medium, the primary medium of choice for co-culturing cryoheps and hiPSC-EHTs. This ability to induce CYPs was also observed in the InnoCell culture. These results suggest that the HM Dex (-) medium is also useful for evaluating cardiotoxicity via drug–drug interactions.

Hepatocytes cultured on normal plastic plates experience hypoxia [26]. Moreover, primary human hepatocytes require high oxygen levels, approximately 12 times higher than those in HepaG2 cells and five times higher than those in HepaRG cells [48]. We hypothesized that CYP metabolism may be improved using an InnoCell plate, which features an oxygen-permeable membrane. InnoCell plate-mediated improvement of oxygen supply significantly increased the expression of *CYP3A4* and the activities of CYP1A, CYP2D6, and CYP3A in the HM Dex (-) medium. The change in CYP3A activity due to the improved oxygen supply correlated with changes in expression. However, the changes in CYP1A and CYP2D6 activities due to improved oxygen could not be explained by changes in gene expression, suggesting that improved oxygen supply causes changes at the enzyme level. Niklas et al. found that CYP3A4 activity was correlated with the amount of protein in various media, but no correlation was found between CYP2C9, CYP2C19, and CYP2D6 protein levels and activity [49]. This is consistent with our results. Not only the expression but also the half-life of CYP and cofactors such as NADPH are involved in CYP activity. Improved oxygen supply in InnoCell cultures is likely to enhance aerobic metabolism, suggesting that changes in NADPH production may be involved in enhancing CYP activity through improved oxygen supply.

The insights gained in this study on HCMM lay the groundwork for the construction of co-culture systems for hepatocytes and cardiomyocytes using MPS, such as the KIM-plate. However, further studies using the HM Dex (-) medium are necessary to optimize cardiotoxicity evaluations via hepatic drug metabolism in co-culture settings. Selecting an appropriate co-culture medium is an important consideration in cardiotoxicity evaluation via hepatic metabolism using MPS. Among the media evaluated, HM Dex (-) emerged as the best HCMM and the most promising candidate medium for co-culturing cryoheps and hiPSC-EHT. In the future, we plan on co-culturing cryoheps and iPSC-EHT in HM Dex (-) medium using the KIM-plate. It is important to consider cell–cell interaction in co-culture because secretions from each cell type can affect the function of other cells. In this study, the cardiotoxicity of terfenadine and paliperidone was evaluated in mono-culture of hiPSC-EHT using HM Dex (-) medium as described in a previous report (S3 Fig) [36, 37], but these evaluations have not been conducted in co-culture of cryoheps and hiPSC-EHT. Future work will focus on evaluating the cardiotoxicity of several drugs whose cardiotoxicities are known in the co-culture of cryoheps and hiPSC-EHT to further verify the utility of HM Dex (-) medium for cardiotoxicity evaluation via hepatic metabolism. Furthermore, we aim to investigate the correlation between our *in vitro* findings and *in vivo* data. Through these studies, we hope to contribute to reducing the number of drugs withdrawn from the market owing to cardiotoxicity, as well as minimizing reliance on animal testing, cutting development costs, and shortening development time.

## Supporting information

S1 Fig Relative expression levels of 17 myocardial-specific genes in human iPS cell-derived engineered heart tissues (hiPSC-EHTs) cultured in three candidate media compared to that in the left ventricle. hiPSC-EHTs were cultured in iCell cardiomyocyte maintenance medium for 3 weeks and then in the EHT medium for over 1 week. Thereafter, hiPSC-EHTs were cultured in the three candidate media for 72 h. The graph bar shows the average mean, and the error bar shows the standard error (n = 4).
(TIF)

S2 Fig Two dimensional plot of the eigenvalues in the first and second components of the Principal Component Analysis (PCA). PCA was conducted based on the log2 values (relative expression levels compared with the left ventricle) of the 17 genes using MetaboaAnalyst 6.0. 2D plot of eigenvalues in the first component and second component. (https://www.metaboanalyst.ca/MetaboAnalyst/ModuleView.xhtmL).
(TIF)

S3 Fig Cardiotoxicity evaluation to paliperidone or terfenadine of human iPS cell-derived engineered heart tissues (hiPSC-EHTs). hiPSC-EHTs were cultured in HM Dex (-) medium. hiPSC-EHTs were cumulatively exposed to paliperidone or terfenadine and movies were recorded 15 min after exposure. Movie images were analyzed using SI8000 or MUSCLEMOTION software. A) Changes in contraction waveform to paliperidone (0, 0.1 and 0.3 mM). B) Changes in contraction length and C) contraction waveform to 1 and 3 mM terfenadine. Arrows indicate arrythmia waveform.
(TIF)

S4 Fig *IL6* expression in human cryopreserved hepatocytes (cryoheps). Cryoheps were cultured in HM medium, or HM Dex (-) medium on polystyrene plates for 72 h from the day of seeding. RNA was collected from the cells at the endpoint and analyzed using qPCR. The graph bar shows the average mean of the relative expression levels compared with the human liver, and the error bar shows the standard error (n = 3).
(TIF)

S1 Table Candidate common media for the co-culture of human cryopreserved hepatocytes and human iPS cell-derived engineered heart tissues. (XLSX)

S2 Table Primer and probe sets used to evaluate human cryopreserved hepatocytes. (XLSX)

S3 Table Primers used to evaluate human iPS cell-derived engineered heart tissues. (XLSX)
