# Supplementary figures and images for: Examination of common culture medium for human hepatocytes and engineered heart tissue: Towards an evaluation of cardiotoxicity associated with hepatic drug metabolism in vitro
Source: PLoS One. 2024 Dec 23;19(12):e0315997. doi: 10.1371/journal.pone.0315997 (PMC11666010; doi:10.1371/journal.pone.0315997)

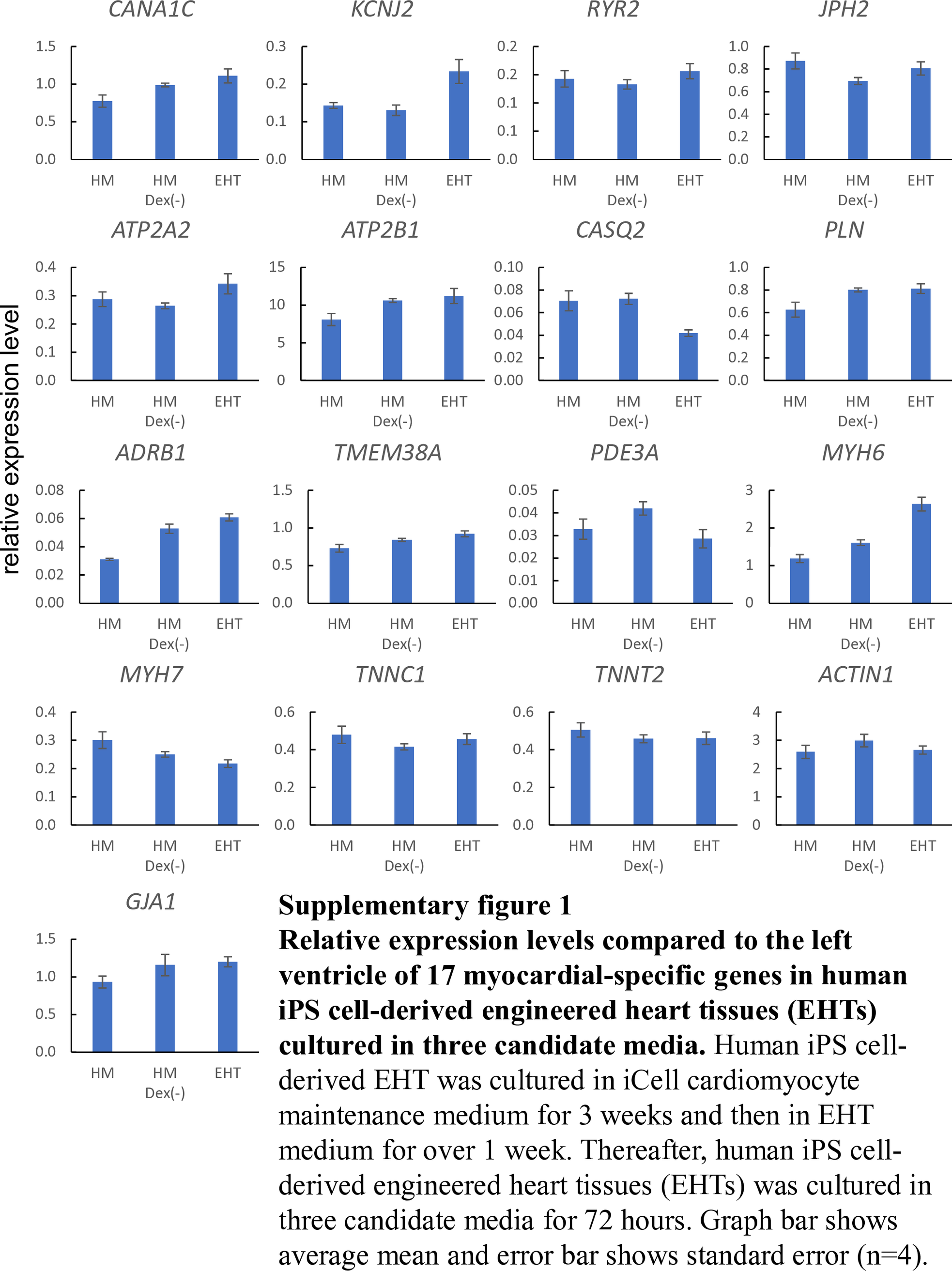

Supplement: S1 Fig — hiPSC-EHTs were cultured in iCell cardiomyocyte maintenance medium for 3 weeks and then in the EHT medium for over 1 week. Thereafter, hiPSC-EHTs were cultured in the three candidate media for 72 h. The graph bar shows the average mean, and the error bar shows the standard error (n = 4). (TIF) [file pone.0315997.s001.tif]

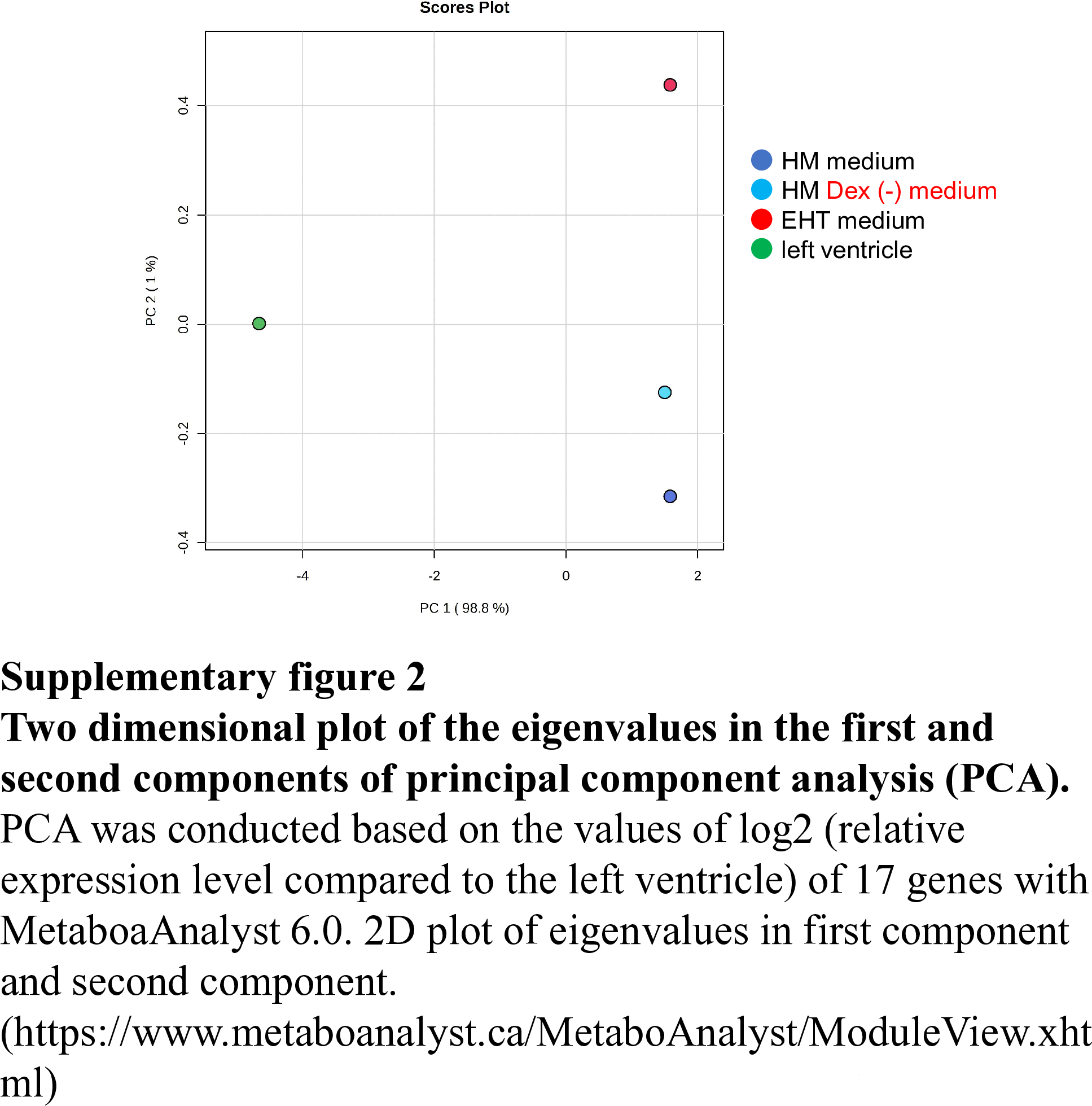

Supplement: S2 Fig — PCA was conducted based on the log2 values (relative expression levels compared with the left ventricle) of the 17 genes using MetaboaAnalyst 6.0. 2D plot of eigenvalues in the first component and second component. (https://www.metaboanalyst.ca/MetaboAnalyst/ModuleView.xhtmL). (TIF) [file pone.0315997.s002.tif]

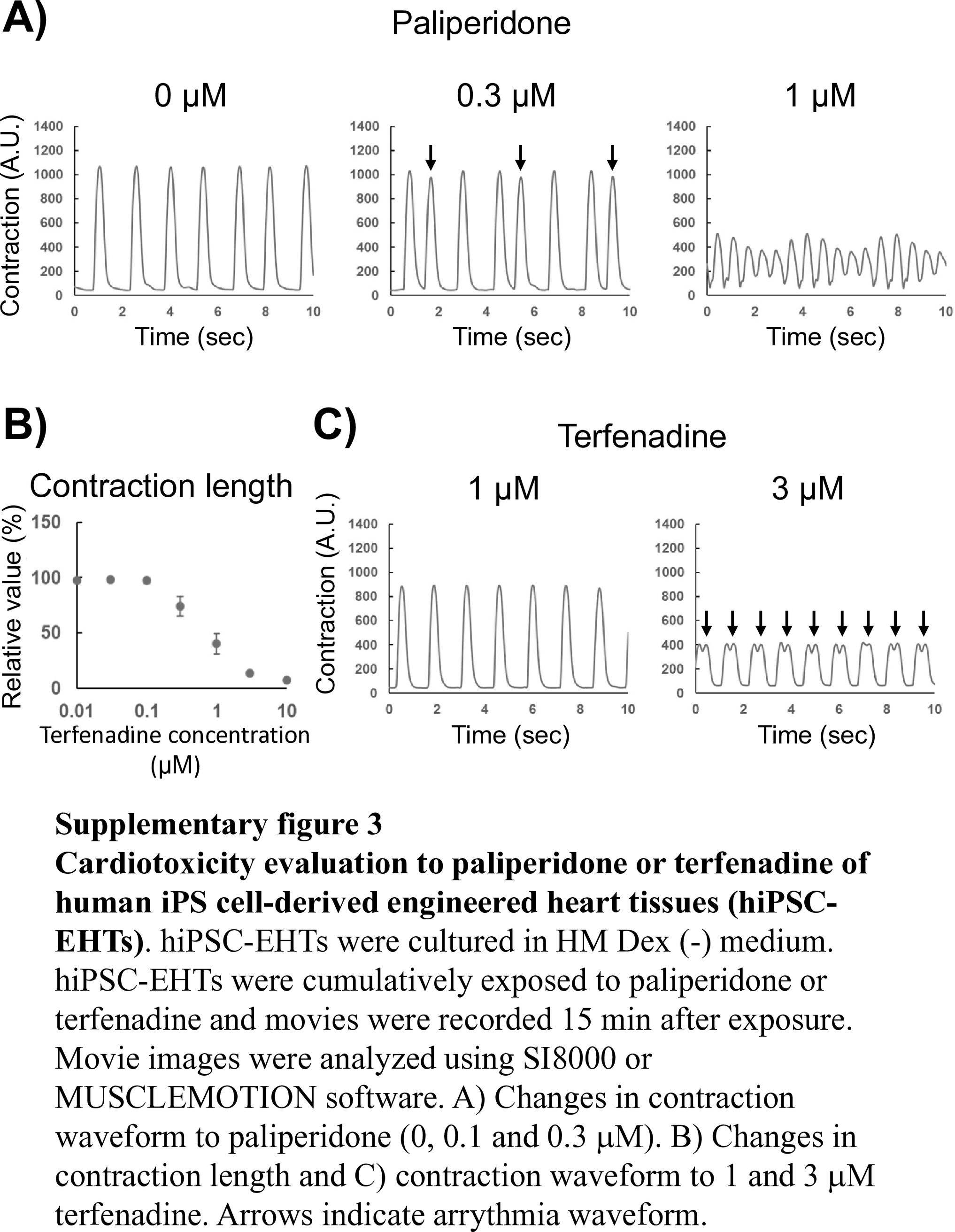

Supplement: S3 Fig — hiPSC-EHTs were cultured in HM Dex (-) medium. hiPSC-EHTs were cumulatively exposed to paliperidone or terfenadine and movies were recorded 15 min after exposure. Movie images were analyzed using SI8000 or MUSCLEMOTION software. A) Changes in contraction waveform to paliperidone (0, 0.1 and 0.3 mM). B) Changes in contraction length and C) contraction waveform to 1 and 3 mM terfenadine. Arrows indicate arrythmia waveform. (TIF) [file pone.0315997.s003.tif]

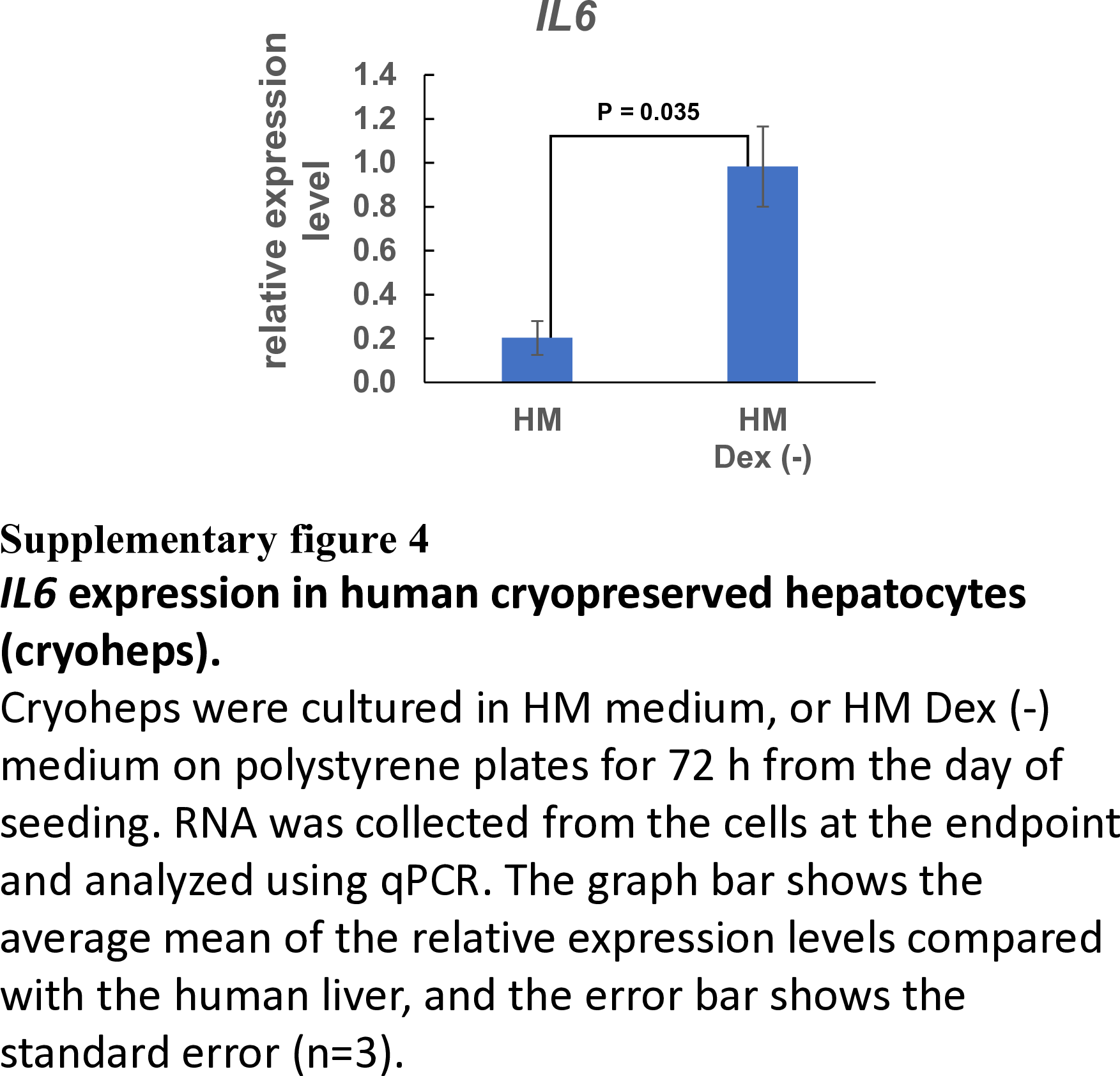

Supplement: S4 Fig — Cryoheps were cultured in HM medium, or HM Dex (-) medium on polystyrene plates for 72 h from the day of seeding. RNA was collected from the cells at the endpoint and analyzed using qPCR. The graph bar shows the average mean of the relative expression levels compared with the human liver, and the error bar shows the standard error (n = 3). (TIF) [file pone.0315997.s004.tif]
